# In situ plasmonic optical fiber detection of the state of charge of supercapacitors for renewable energy storage

**DOI:** 10.1038/s41377-018-0040-y

**Published:** 2018-07-11

**Authors:** Jiajie Lao, Peng Sun, Fu Liu, Xuejun Zhang, Chuanxi Zhao, Wenjie Mai, Tuan Guo, Gaozhi Xiao, Jacques Albert

**Affiliations:** 1 Guangdong Key Laboratory of Optical Fiber Sensing and Communications, Institute of Photonics Technology, Jinan University, Guangzhou, Guangdong 510632 People’s Republic of China; 2 Department of Physics, Siyuan Laboratory, Guangdong Provincial Engineering Technology Research Center of Vacuum Coating Technologies and New Energy Materials, Jinan University, Guangzhou, Guangdong 510632 People’s Republic of China; 3 Department of Electronics, Carleton University, Ottawa, K1S 5B6 Canada; 4 Advanced Electronics and Photonics Research Center, National Research Council of Canada, Ottawa, K1A 0R6 Canada

## Abstract

In situ and continuous monitoring of electrochemical activity is key to understanding and evaluating the operation mechanism and efficiency of energy storage devices. However, this task remains challenging. For example, the present methods are not capable of providing the real-time information about the state of charge (SOC) of the energy storage devices while in operation. To address this, a novel approach based on an electrochemical surface plasmon resonance (SPR) optical fiber sensor is proposed here. This approach offers the capability of in situ comprehensive monitoring of the electrochemical activity (the electrode potential and the SOC) of supercapacitors (used as an example). The sensor adopted is a tilted fiber Bragg grating imprinted in a commercial single-mode fiber and coated with a nanoscale gold film for high-efficiency SPR excitation. Unlike conventional “bulk” detection methods for electrode activity, our approach targets the “localized” (sub-μm-scale) charge state of the ions adjacent to the electrode interface of supercapacitors by monitoring the properties of the SPR wave on the fiber sensor surface located adjacent to the electrode. A stable and reproducible correlation between the real-time charge–discharge cycles of the supercapacitors and the optical transmission of the optical fiber has been found. Moreover, the method proposed is inherently immune to temperature cross-talk because of the presence of environmentally insensitive reference features in the optical transmission spectrum of the devices. Finally, this particular application is ideally suited to the fundamental qualities of optical fiber sensors, such as their compact size, flexible shape, and remote operation capability, thereby opening the way for other opportunities for electrochemical monitoring in various hard-to-reach spaces and remote environments.

## Energy storage: Watching supercapacitors at work

An optic fiber system developed by researchers in China and Canada can peer inside supercapacitors and batteries to observe their state of charge. Renewable energy sources are naturally inconsistent, and so require new energy storage technologies. Supercapacitors offer rapid charging and long-term storage, but it is important to be able to monitor their working state. To tackle this issue, a team including Tuan Guo and Wenjie Mai at Jinan University adapted a standard telecommunications optic fiber to act as a grating, with a very thin gold coating that supports electron oscillations called surface plasmons. When the researchers installed their fiber in a supercapacitor they found that the optical properties of the surface plasmons changed depending on the supercapacitor’s state of charge, offering a unique, low-cost method for real-time monitoring of energy storage devices in operation.

## Introduction

Renewable energy produced by the sun, ocean, and wind has been considered to be a cleaner technology than widely used fossil fuel-based energy sources. However, it is impossible to supply these forms of renewable energy steadily and continuously. Therefore, energy storage devices, such as supercapacitors and batteries, are commonly used for irregularly producing clean energy sources. Among these, supercapacitors offer advantages such as fast-charging, high energy density, and long life cycle storage solutions^1–3^. According to the charge storage mechanism, supercapacitors can be generally classified into two types^4–6^. One is the electric double layer capacitor (EDLC), which stores energy through charge absorption/desorption on the surface of an electrode^7–9^. The other is the pseudocapacitor, which stores energy by electron transfer between an electrode and an electrolyte through electrochemical reactions, i.e., the fast and reversible redox reactions occurring on the surface of the electrodes^10–14^.

In order to better understand the operation mechanisms and improve the charge efficiency of supercapacitors, continuous monitoring of the working state of supercapacitors is necessary. For instance, transmission electron microscopy (TEM) has been widely used to observe dynamic processes within batteries during charging and discharging^15–19^. Additionally, Cui’s group proposed cryo-electron microscopy (cryo-EM) to monitor the atomic structure and interfaces of the battery materials in order to obtain a comprehensive understanding of the mechanisms of energy storage^20^. However, both methods employ expensive and bulky instrumentation that are not practical for general applications or routine use. The more common supercapacitor monitoring methods (like cyclic voltammetry (CV) and galvanostatic charge–discharge method) are based on off-line measurements of current and voltage data to yield a “calculated capacity” that may not reflect the exact and instantaneous state of charge (SOC) of the supercapacitors. Furthermore, the reliability of those results can deteriorate over repeated cycling processes^21^. Therefore, the development of easy-to-use, practical methods for the measurement of the SOC in real time becomes essential for field monitoring of supercapacitors^22^.

Electrochemical surface plasmon resonance (EC-SPR) offers a promising way to simultaneously explore optical and electrochemical properties of the chemicals involved in redox reactions at surfaces. Optically excited and probed plasmon waves provide an increased sensitivity to chemical changes due to the strong localization of electromagnetic energy in the layer immediately adjacent to the metal surface^23^. Any perturbation in this layer, such as the bonding of target analytes to receptor molecules, modifies the local complex refractive index and therefore the plasmon phase velocity and attenuation distance^24^. Recent studies in this field include plasmonic imaging of surface charge density^25^, single nanoparticle electrochemistry^26^, and plasmonic monitoring of the charge status of small molecules^27, 28^. However, these methods are typically carried out using free space optics and a bulky glass prism, which prevent their applications for in situ monitoring of SOC of supercapacitors.

Herein, we report the first application of in situ monitoring of the electrochemical activity in supercapacitors using plasmonic optical fiber sensors. We demonstrate that there is an intrinsic relationship between the SOC of supercapacitors and the measured changes in the optical properties of surface plasmon waves on the fiber surface. The optical fiber sensor demonstrated here consists of a tilted fiber Bragg grating (TFBG) imprinted in the core of a commercial single-mode fiber typical of those used in telecommunications, provided with an additional nanometer-scale gold coating to support plasmon waves^29^. Such plasmonic optical fiber sensor is cost-effective and much simpler to interrogate compared to the well-established bulky prism configurations discussed above. Its compact size makes it possible to be inserted into various hard-to-reach environments for in situ detection either as a hand-held probe or as a set of remotely operated devices fixed at various locations in the supercapacitor along a fiber-optic cable. Another particular advantage of the TFBG platform is a means to mitigate temperature cross-sensitivity: as will be demonstrated, the optical spectrum of the devices used contains a feature corresponding to light remaining in the core of the fiber (the “Bragg resonance”) and thus is inherently insensitive to changes external to the fiber, apart from temperature and strain. Strain effects can be eliminated by suitable packaging while temperature effects can be calibrated out by using the Bragg resonance as a thermometer (a widely used application of FBGs)^29^. For chemical changes immediately adjacent to the metal surface of the TFBG, however, the associated modification of the complex refractive index has a very strong impact on the phase velocity and attenuation of the SPR, an effect that is clearly reflected in the measured transmission of specific, high Q-factor resonances of the device. The sensor fabrication process, i.e., UV-light grating-inscription and surface nanometer-sized coating, does not affect the structural integrity of the fiber, hence ensuring the sensor robustness and reproducibility. Finally, the relationship between the sensor response and the SOC of the supercapacitors is found to be highly reproducible. The information provided by this kind of sensor will be beneficial to understanding and evaluating the performance of supercapacitors in active service.

## Results

### Optical SPR response of TFBGs to the charging/discharging of supercapacitors

**Fig. 1:**
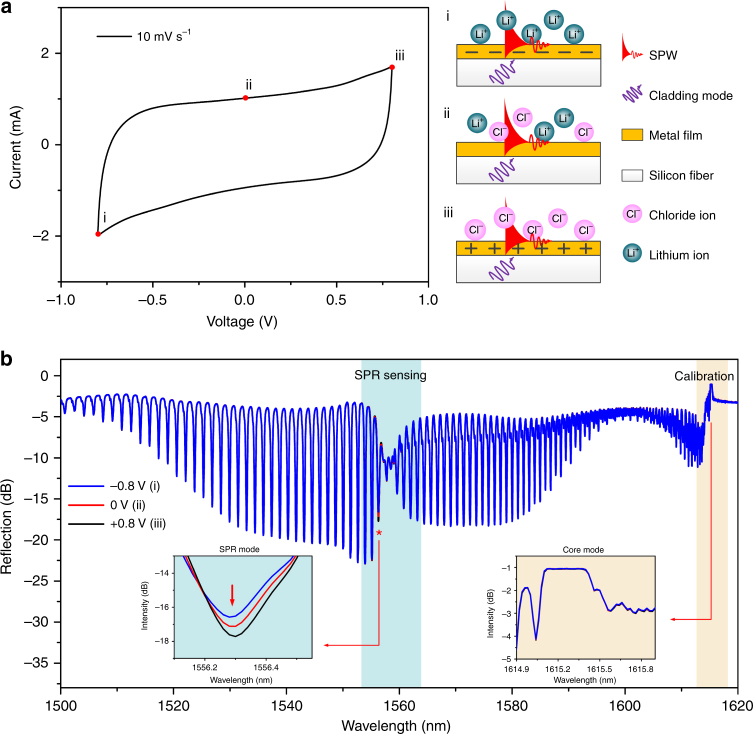
Electrochemical surface-plasmon-resonance sensing principle and experimental demonstration with an gold-coated TFBG optical fiber sensor. **a** CV response of the supercapacitor during a polarizing cycle including positive and negative polarities, and the resulting electronic polarizations of Au film: (i and iii) two opposite polarizations at −0.8 V and +0.8 V; (ii) neutral state (0 V). **b** Spectral response of P-polarized input, gold-coated 18° TFBG for the different electronic polarization states of the electrode; enlarged view (blue inset) of the spectral change of the SPR coupled cladding mode used for monitoring (marked by a red asterisk “*”); and (yellow inset) enlarged view of the spectrum near the core mode (Bragg) reference resonance

As discussed above, the position and amplitude of the SPR in the spectrum of a TFBG are directly related to the complex permittivity close to and in the metal film itself. Therefore, when the gold-coated optical fiber sensor is closely attached to the surface of the electrode, the change of charge density and ions distribution (corresponding to the SOC of supercapacitors) around the electrode can be directly monitored by reading the changes of the SPR spectrum of the sensor. Figure 1a (left) presents the CV curve measured in one cycle. The electronic polarization state of the metal film at three potential points, −0.8 V, 0 V, and +0.8 V, is considered. At point iii (+0.8 V), a large quantity of negative charges was attracted to the positive electrode. Thus, the gold film over the fiber was electronically polarized under the effect of interface capacitance in the supercapacitor. At point ii (0 V), the gold film was at the steady state and the supercapacitor was not charged. Finally, at point i (−0.8 V), the charging polarity on the electrode surface turns to positive so that an opposite electronic polarization would appear. During the CV curve measurements, optical SPR spectra were recorded simultaneously, and these results are shown in Fig. 1b. In such spectra, the “best” resonances to monitor are those within the SPR attenuation region (blue-shaded in Fig. 1b) but not so attenuated as to become ill-defined and broadened. The resonance near 1556.3 nm (marked with an asterisk in Fig. 1b) was chosen here because it is clearly attenuated by the transfer of energy to the plasmon, but it remains well-defined with a full width at half maximum on the order of 0.1 nm, yielding a resonance Q-factor of 15,000. As a first indication of the sensor response, the left inset of Fig. 1b shows a clear correspondence between the amplitude of the resonance and the electronic polarization of the gold layer: the resonance becomes deeper (shallower) for positive (negative) polarities, relative to the zero potential state. In addition, the right inset provides a zoomed-in view of the Bragg resonance (yellow shaded) during polarity cycling, demonstrating that the core mode is totally insensitive to the electrochemical changes, and therefore, its spectrum can be used as a power and wavelength reference to remove the impact of any system instability (or in the case of the wavelength, it acts as an in situ thermometer).

**Fig. 2:**
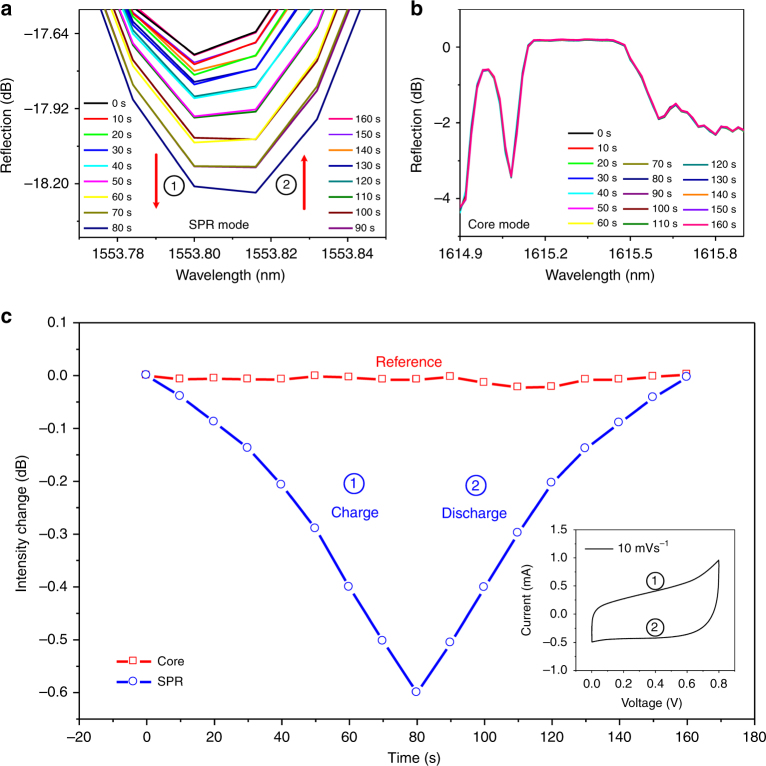
Detailed charging and discharging of the supercapacitor from 0 to 0.8 V. **a** Spectral response of the selected SPR resonance (the red arrow indicates the variation trend of the SPR spectral feature with time) and (**b**) unchanged core Bragg resonance. The electrochemical curve and the optical response are synchronously recorded. **c** Intensity change of the selected SPR resonance (blue dot) and the core Bragg resonance (red block) corresponding to one cycle of the CV curve of the supercapacitors at a scan rate of 10 mV s^−1^ (shown in the inset). The symbol ① represents the process of charging. Conversely, the symbol ② represents the process of discharging

Figure 2 presents a more detailed investigation during a rapid CV measurement of a positive polarity cycle of the supercapacitor from 0 to +0.8 V over just 1 min. Figure 2a, b shows the SPR and Bragg resonances at various points during cycling while Fig. 2c summarizes the amplitudes of the resonances as a function of time. The SPR spectral response is clearly only related to the SOC of the supercapacitor regardless of whether the supercapacitor is charging or discharging (the Bragg resonance remains unaffected). It is now obvious that by correlating the spectral results to the recorded electrochemical curve (the CV measurement over one cycle shown in the inset of Fig. 2c), the real time in situ information of the SOC of supercapacitors can be obtained, as will be demonstrated.

### SOC monitoring

**Fig. 3:**
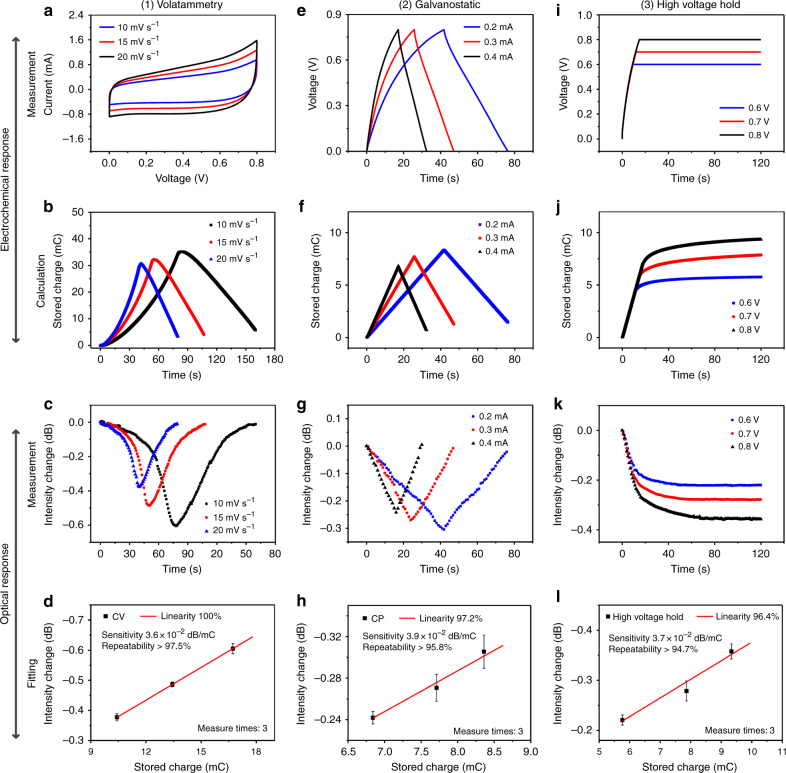
Comprehensive electrochemical measurements and the corresponding SPR response spectra. **a** CV curves for the supercapacitor at scan rates of 10, 15, and 20 mV s^−1^, (**b**) stored charge calculated from CV curves, (**c**) the corresponding sensor SPR transmitted intensity change, and (**d**) linear fit of the change of sensor SPR transmitted intensity vs. the maximum stored charges, where each data point is the average of three CV tests. **e** GCD curves at currents of 0.2, 0.3, and 0.4 mA, (**f**, **g**) the corresponding stored charge and SPR response, and (**h**) linear fit of the averages from three GCD tests; (**i**) galvanostatic charge and voltage holding (GCVH) test, (**j–l**) similar data extracted as in (**b**–**d**) and (**f**–**h**)

**Fig. 4:**
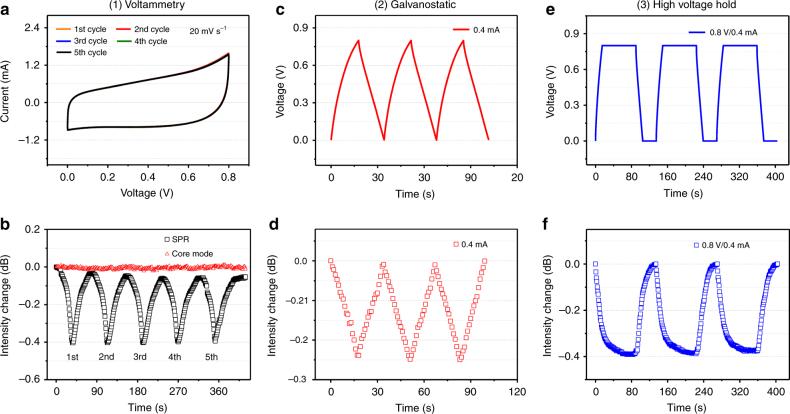
Sensor responses in the charging/discharging cycling tests. **a** Five cycles of CV data and (**b**) the corresponding sensor SPR transmitted intensity; (**c**, **d**) same as (**a**, **b**) but for 3 cycles of GCD; (**e**, **f**) same, but for 3 cycles of GCVH

In order to verify the suitability of the proposed sensor for the monitoring of the SOC of a supercapacitor, three types of charging/discharging tests were employed: the normal voltammetry test, the galvanostatic charge/discharge test, and the high voltage hold test. The results are presented in Figs. 3 and 4.

CV curves measured at three different scan rates, i.e., 10, 15, and 20 mV s^−1^, were collected, shown in Fig. 3a, while the corresponding SOC was calculated from the current (${C} = \mathop {\int }\nolimits I{\rm d}t$, where *I* is the instant current and *t* is the corresponding time in the CV curves), shown in Fig. 3b. The upper part of the CV curves (Fig. 3a) represents the charging process, in which the ions accumulate around the positive electrode of the supercapacitor and cause an increase of the stored charge. Figure 3a, b confirms that the SOC decreases slightly with the scanning rate increase. This result is opposite to the general belief as both ion movement in the electrolyte and the intercalating process on the electrode take time. The SPR spectra of the developed sensor corresponding to the three voltammetry tests at different scanning rates were recorded. The variations of SPR intensity during the charging and discharging process were calculated and are presented in Fig. 3c. Figure 3d shows the relationship between the maximum stored charge (*C*_max_) and the corresponding SPR intensity variation (ΔSPR) for three repetitions of the measurements at each scan rate. A linear fit of the data indicates a relationship between *C*_max_ (in mC) and ΔSPR (in dB) expressed as ΔSPR=−0.036 × *C*_max_−2.07 × 10^−5^, with a regression coefficient *R*^2^ of 100% and repeatability of 97.5% (1−ΔSPR_error_/ΔSPR_average_). It should be noted that the above SPR intensity variation (ΔSPR) is essentially caused by the charging of the supercapacitor (the electrode), not the charging of the gold on the optical fiber. Figure S2 clearly demonstrates this point by providing the experimental comparison results between the optical fiber SPR responses for supercapacitor monitoring with and without electrochemical capacitive material (MnO_2_) on the surface of carbon fabrics.

Similar phenomena can be observed for the results obtained from the galvanostatic charge–discharge (GCD) tests (Fig. 3e, f). When the high voltage of the charging process is fixed, the stored charge in supercapacitors is determined by the current flowing through the electrode. In this test, three currents were employed, i.e., 0.2, 0.3, and 0.4 mA. When the current is lower, more charges can be gradually accumulated on the surface or in the inner layer of the pseudo-capacitive electrode. Thus, the stored charge at the current of 0.2 mA reaches the highest value among the three different currents. Additionally, the intensity change in the SPR response is higher because the corresponding stored charge is larger (Fig. 3g), strongly supporting the result of the CV measurement. A linear fit of the relationship between *C*_max_ and ΔSPR in this test, also based on three repetitions, results in the following: ΔSPR = −0.039 × *C*_max_ + 2.75 × 10^−2^ (Fig. 3h), with *R*^2^ = 97.2% and a repeatability of 95.8%.

In the high voltage hold test, the sensor was subjected to a galvanostatic charge test with different ending voltages and then retained this voltage for 2 min. The testing results are shown in Fig. 3i–l. It can be noted from Fig. 3i, j that the stored charge increases with the holding voltage between 0.6 and 0.8 V. For this case, the fitting result between *C*_max_ and ΔSPR is ΔSPR = 0.037 × *C*_max_−4.68 × 10^−3^, with *R*^2^ = 96.4% and a repeatability of 94.7%.

This method can also be used under faster charging and discharging speeds. As presented in Figures S3 and S4, in situ CV and GCD measurements at much higher charging/discharging currents (2–4 mA) and faster scan speeds (200–400 mV s^−1^) were performed (both are 10 times higher than for the data in Fig. 3). These results show that under the CV and GCD tests, our system can effectively work at higher charging/discharging speeds. By calculating from the CV and GCD curves, the maximum SPR intensity variation corresponding to the maximum charge stored in the supercapacitor under different scan rates and currents was linearly fitted and presents excellent linearity and similar sensitivities (Figure S4). It should be noted that the value of sensitivity is a little lower than that under slow scan rates and lower current, as seen in Fig. 3. This slight difference should be calibrated for different cases of in-field applications. The present interrogation speed is highly limited by the optical spectrum analyzer (because of interrogation based on spectrum acquisition). This problem can be definitely solved by using a real-time interrogation scheme based on power measurement in a narrow band of the optical spectrum^30^. In this case, a tunable laser (TLS) can be used as a source instead of a broadband source, together with a photodiode (PD) as detector and an analog-to-digital converter (A/D), to obtain the desired data (to replace the optical spectrum analyzer). The function of the TLS is to probe the transmission at the wavelength of the most sensitive mode of the fiber grating (the SPR mode here), determined by initial calibration with a spectrum analyzer. This technique relies on the principle of edge filtering so that the optical power change is produced as a result of the wavelength shift of the mode with respect to the fixed wavelength of the laser source.

The results in Fig. 3 indicate that the charge stored in the supercapacitor can be inferred from transmission changes at the SPR wavelength of the TFBG spectrum, regardless of the method used to determine the charge/ΔSPR relationship, estimated to be 3.7(±0.15) × 10^−2^ dB mC^−1^ (however, the value of 3.6 × 10^−2^ dB mC^−1^ obtained by voltammetry appears to be more reliable in view of the fitting results). So, once a particular sensor is calibrated, it can be used to follow the SOC in a supercapacitor during its life cycle without having to interrupt operations to carry out electrical testing.

In order to further support the statement just made, repeated charging/discharging cycling tests were performed. In Fig. 4a, b, the CV curves of 5 cycles of voltammetry at a scan rate of 20 mV s^−1^ and the corresponding sensor SPR intensity are presented. Similar measurements were carried out for GCD tests at 0.4 mA of current (Fig. 4c, d) and GCVH tests at 0.8 V and 0.4 mA (Fig. 4e, f). In all cases, little or no decay in the SPR response can be observed over the cycles presented (with the exception of a small drift of the zero charge SPR transmission intensity level in the CV test, with no apparent impact on the measurement of the maximum charged state near −0.42 dB).

## Discussion

In this work, a novel method for the in situ monitoring of the capacity stored in supercapacitors was proposed for the first time. The method is based on a plasmonic TFBG sensor that is attached to one of the electrodes of the supercapacitor under testing. It was found that the SPR spectrum of the developed sensor clearly follows the charging and discharging processes of the supercapacitor. The CV, GCD, and GCVH tests demonstrated that the SPR response of the developed sensor can be correlated quantitatively to the charge stored in the supercapacitor. Furthermore, the SPR response to stored charge changes was demonstrated to be stable over several cycles of charge and discharge. As a result, the proposed device provides a new approach in the study of the charging/discharging process of supercapacitors. In addition, since the TFBG sensor proposed is made from silica glass and gold metal and since it operates exclusively with light signals carried to and from the sensor by standard telecommunication-grade optical fibers, with suitable packaging, it is expected to be robust enough to remain attached to the supercapacitor electrodes in operation and (following an initial calibration) to provide real-time remote monitoring of the SOC of supercapacitors used for power supply regulation from renewable energy sources.

## Materials and methods

### Sensing system

**Fig. 5:**
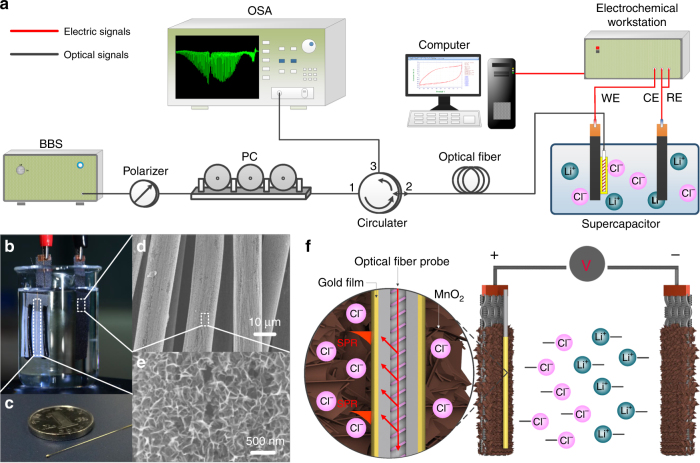
Electrochemical surface-plasmon-resonance sensing principle and experimental demonstration with an gold-coated TFBG optical fiber sensor. **a** Experimental setup of a plasmonic fiber-optic sensing system for monitoring the SOC of supercapacitors. **b** Photographs of the configuration for the supercapacitor and (**c**) gold-coated fiber-optic sensing probe. SEM images of (**d**) the MnO_2_ electrode and (**e**) the corresponding magnified image. **f** Schematic of the measurement of the charge–discharge process of supercapacitors by a plasmonic gold-coated TFBG fiber-optic sensor

The all-fiber-coupled EC-SPR fiber-optic sensing system employed is shown in Fig. 5a and comprises a broadband light source (BBS) with bandwidth from 1250 to 1650 nm, a polarizer, a polarization controller (PC), a circulator, a plasmonic optical fiber sensing probe, and an optical spectrum analyzer (OSA). An electrochemical workstation is used for performing conventional electrochemical measurements and collecting supercapacitor data to be correlated to the optical measurements. A computer was used to collect simultaneous data from both systems as the supercapacitor cycled through charge and discharge. Figure 5b presents the detailed configuration of the measurement system containing a supercapacitor (two MnO_2_@carbon fabric electrodes in liquid electrolyte, with an area of 3 cm^2^ soaked in electrolyte) and a plasmonic fiber-optic sensing probe coated with a nanometer-scale gold film. The entire plasmonic fiber-optic sensing probe is very compact, with a size of 30 mm in length and 125 μm in diameter (Fig. 5c). The probe can be tightly attached to any electrode of the supercapacitor.

### Supercapacitor

The scanning electron microscopy (SEM) images in Fig. 5d, e show the morphology of the uniform MnO_2_ nanosheets stacked over the surface of the carbon fiber fabric. A solution containing 0.1 M MnAc_2_ and 0.1 M NaAc was used to provide Mn ions. The carbon fabric (area of 1 × 3 cm^2^) was soaked into the Mn ions solution and a constant current density of 1 mA cm^−2^ was applied. The MnO_2_ was synthesized by an electrodeposition method that used a three-electrode system with Ag/AgCl as the reference electrode and Pt as the counter electrode. The whole synthesis process was achieved in 5 min.

Figure 5f shows the configuration of the supercapacitor and plasmonic optical fiber sensor. The supercapacitor used here is a pseudo-capacitor. It stores electrical energy on the basis of an electrical double layer effect over the material surface and fast bi-dimensional redox reactions in a very thin electrode surface layer. Sensing is based on the fact that the plasmon waves excited by the grating in the core of the optical fiber probe have a high percentage of their propagating power localized in a 1-μm-thick layer above the metal surface. Therefore, when the fiber probe is positioned in the layer where the redox reactions occur, there are observable changes in the SPR optical spectrum.

### Fabrication of TFBG-based SPR optical fiber sensor

**Fig. 6:**
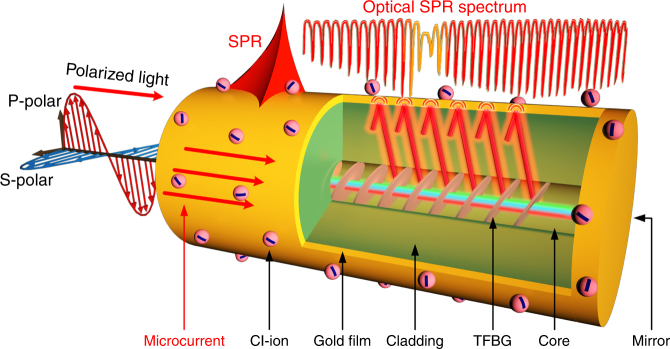
Electrochemical surface-plasmon-resonance sensing principle and experimental demonstration with an gold-coated TFBG optical fiber sensor. Sketch of the configuration of a plasmonic optical fiber sensor for in situ monitoring of supercapacitors

TFBG probes were manufactured in commercial photosensitive single-mode fiber (provided by Corning Incorporated) using a well-established technique described in ref. ^27^. Specifically, the TFBG was manufactured using the phase-mask technique by shining UV light pulses from an excimer laser (at a wavelength of 193 nm and with a power of 30 mJ per pulse) onto the surface of a bare fiber, after passing through the diffractive mask where the desired grating pattern was etched. In this manner, a corresponding periodic modulation of the refractive index is formed in the fiber core. Contrary to the case for standard fiber Bragg gratings, the planes of the refractive index modulation were written with a pre-defined tilt relative to the longitudinal axis of the fiber (see Fig. 6, where the grating in the fiber core is colored in pink). The tilt of the grating is an important parameter that determines which set of cladding modes is excited: here, an 18 degree angle is chosen to maximize coupling to cladding modes that are suitable to transfer energy to surface plasmons in the aqueous solutions used for electrolytes in supercapacitors.

A 50-nm-thick gold layer of high surface quality was deposited on the above TFBG by sputtering as follows. First, a 2–3-nm buffer layer of chromium is deposited on the optical fiber surface to promote adhesion between the fiber and the gold. Second, gold is sputtered on top of the chromium while the optical fiber is rotated along its axis. This process ensures that the gold layer is uniform around the fiber, which helps in achieving clear SPR effects. Finally, the coated fiber is annealed for 3 h at a temperature of 300 °C so that the gold coating has the desired morphology and is robust enough for sensing applications^31^.

The responses of plasmonic TFBG devices are normally observed in transmission, which requires access to the sensor from both sides. For applications in small areas and, in particular, for this work, it is desirable to have single-ended sensors located close to the end of a fiber, so that it can be inserted into tight spaces. Therefore, an additional (thicker) gold coating is deposited on the end of the fiber, cut a few mm downstream from the TFBG, to act as a broadband mirror with >90% reflectivity, which enables interrogation of the sensor in reflection. In the reflection measurement method, the Bragg resonance (reflection of the core mode upon itself, used for temperature compensation) appears as a spectral peak while cladding mode resonances appear as narrow troughs in the broadband reflection from the end mirror. Figure 6 shows a cartoon representation of a typical sensor packaged for this work. This configuration also ensures that the sensor is strain free when a single attachment point is used to fix the sensor to the electrode surface.

### Principle and characteristics of TFBG-based SPR optical fiber sensors

Surface plasmon polaritons (SPPs) are near-infrared or visible-frequency electromagnetic waves that travel along a metal–dielectric or metal–air interface and decay exponentially away from the interface. The evanescent field of a fiber cladding mode can tunnel through a thin metal coating and transfer energy to an SPP wave of the outside interface of the metal when two necessary conditions are satisfied: (1) the propagation constant of the cladding mode equals that of the SPP for that particular combination of metal and surrounding medium; and (2) the polarization of the light must be perpendicular to the metal surface, i.e., TM like^31, 32^. Only a small subset of the cladding modes of any fiber can meet these conditions.

The propagation constant *β*_SPP_ of SPP is expressed as:

1 $$\beta _{{\rm SPP}} = \frac{\omega }{c}\sqrt {\frac{{\varepsilon _{\rm m}\varepsilon _{\rm s}}}{{\varepsilon _{\rm m} + \varepsilon _{\rm s}}}}$$

where *c* is the speed of light in vacuum, *ω* is the angular frequency of the light, and *ε*_m_ and *ε*_s_ are the complex relative permittivities of the metal film and the surrounding material adjacent to the metal interface where the SPP is located, respectively.

On the other hand, the propagation constants *β*_clad,*i*_ of cladding modes (labeled by the subscript *i*) in a standard fiber with a cladding diameter on the order of 100 times the wavelength can take a large, closely spaced set of values, and the associated fields have widely different polarization properties. The phase-match condition between propagation constants can be expressed as

2 $$\beta _{{\rm SPP}} = \beta _{{\rm clad},i} = 2\pi N_{{\rm clad},i}^{{\rm eff}}{\mathrm{/}}\lambda$$

where the last equality introduces $N_{{\rm clad},i}^{{\rm eff}}$ which is defined as the effective index of the *i*^th^ cladding mode at wavelength *λ*^29^.

**Fig. 7:**
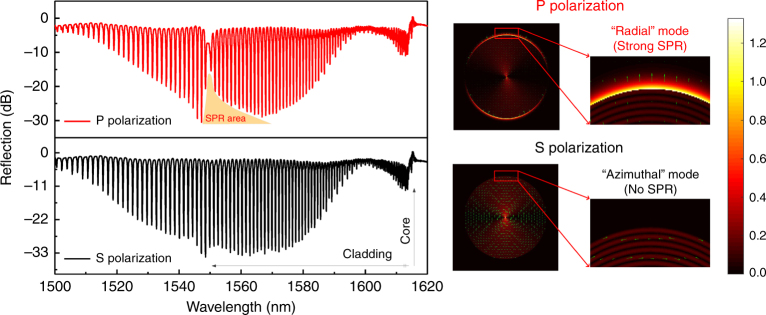
(Left) Reflection spectra of a mirror-ended TFBG optical fiber coated with 50 nm of gold and immersed in water. P-polarized incident light showing SPR near 1550 nm (red curve) and S-polarized incident light (black curve, no SPR observed); (right) Simulated electric mode field profiles for two neighboring high-order cladding modes: modes excited by P-polarized core mode input light have electric fields that are oriented predominantly radially at the boundary (upper), while modes excited from S-polarized input have predominantly tangential electric fields around the fiber cladding boundary (bottom). The color scale reflects the magnitude of the electric fields and the arrows their orientation. The transfer of energy from cladding mode to a surface plasmon shows up as a bright ring around the fiber cladding for P-polarized input

Finally, the phase-matching condition can be observed and measured with great accuracy from the transmission spectrum of a fiber grating because of the one-to-one relationship between effective indices (hence propagation constants) of modes and their resonance wavelengths in the spectrum, expressed by the following additional phase-matching rule:

3 $$\lambda _{{\rm clad},i} = \left( {N_{{\rm clad},i}^{{\rm eff}} + N_{{\rm core}}^{{\rm eff}}} \right){\mathrm{\Lambda }}$$

where $N_{{\rm core}}^{{\rm eff}}$ is the effective index of the input core mode and Λ is the period of the grating (measured along the fiber axis, i.e., not equal to the distance between the tilted grating planes). As can be seen in the measured spectra in Fig. 7 (left), there are many such resonances corresponding to the set of modes supported by the relatively large cladding. A first discrimination between modes is provided by using polarized input core mode light. It was demonstrated that with input light polarized parallel to the inclination plane of the grating (P-polarized), the excited cladding modes have electric fields polarized radially at the surface of the cladding and can thus excite surface plasmons, while the orthogonal input polarization (S-polarized) excites tangentially polarized cladding modes that cannot couple to plasmons^33^. This is clearly demonstrated in Fig. 7 by the fact that only the P-polarized spectrum (red curve) shows a characteristic attenuation of the cladding mode resonance amplitudes for wavelengths near 1550 nm (indicating that power has been “lost” or transferred from the cladding to the surface plasmon) and further by simulations of the electric field profiles for the two cases. The measured S-polarized spectrum (black curve) shows no such attenuation.

Having identified plasmon-coupled resonances in the spectrum at specific wavelengths, Eqs. 1–3 provide a direct link between these wavelengths and the permittivity of the medium just above the metal layer (*ε*_s_). If that permittivity changes, the corresponding resonance position and amplitude will change as well^34^. Such changes include the formation of a new material layer on top of the gold, such as in the well-known and widely applied affinity studies for biomolecules and also in the measurements of faradic currents and double layer charging currents in the metal film itself leading to charge density changes in the metal film and modifications of *ε*_m_. The latter phenomenon is commonly named electrochemical surface plasmon resonance (EC-SPR), and it is a powerful tool to study and identify the electrochemical activity of the “surface” and “localized” charge state of the ions adjacent to the electrode interfaces. Simulations similar to those shown in Fig. 7 reveal that up to 70% of the light power of cladding modes phase matched to plasmons propagating as a bound wave in the external medium, while the “normal” evanescent waves of the cladding modes of bare fiber (with the same TFBG inside) can only carry between 2 and 5% of the mode power^33^.

Finally, another factor important in the current device is that it operates in near infrared instead of the more commonly used visible wavelengths for SPR applications. This extends the penetration depth of the fields of plasmon waves from the 200–300 nm range to more than 1 μm, a more suitable distance for monitoring the electrochemical activities of ions just over the surface of the electrodes.

### Data availability

The data that support the findings of this study are available from the corresponding authors upon request.

## Electronic supplementary material

Supplementary material
