# Supplementary material for: In situ plasmonic optical fiber detection of the state of charge of supercapacitors for renewable energy storage
Source: Light Sci Appl. 2018 Jul 11;7:34. doi: 10.1038/s41377-018-0040-y (PMC6106991; doi:10.1038/s41377-018-0040-y)
Supplement: Supplementary file 1 — Supplementary material [file 41377_2018_40_MOESM1_ESM.docx]

**Supporting Information**

*In Situ* Plasmonic Optical Fiber Detection of the State of Charge of Supercapacitors for Renewable Energy Storage

Jiajie Lao^1^, Peng Sun^2^, Fu Liu^1,3^, Xuejun Zhang^1^, Chuanxi Zhao^2^, Wenjie Mai^2^*, Tuan Guo^1^*, Gaozhi Xiao^4^ & Jacques Albert^3^

^1^ Guangdong Key Laboratory of Optical Fiber Sensing and Communications, Institute of Photonics Technology, Jinan University, Guangzhou, Guangdong 510632, PR China

^2^ Siyuan Laboratory, Guangdong Provincial Engineering Technology Research Center of Vacuum Coating Technologies and New Energy Materials, Department of Physics, Jinan University, Guangzhou, Guangdong 510632, PR China

^3^ Department of Electronics, Carleton University, Ottawa K1S 5B6, Canada

^4^ Advanced Electronics and Photonics Research Center, National Research Council of Canada, Ottawa K1A 0R6, Canada

The two first authors contributed equally to this work.

*Corresponding authors: W. Mai ([wenjiemai@gmail.com](mailto:wenjiemai@gmail.com)) and T. Guo ([tuanguo@jnu.edu.cn](mailto:tuanguo@jnu.edu.cn))

**The surface morphology of the gold coating TFBG**

Uniformity of the gold coating on the whole fiber was shown in SEM images in Figure S1. It can be seen that a very uniform nanometric coating on fiber surface was achieved. This is due to the design that during the sputtering process the fiber was rotated along its axis.

**Simulation for SPR as a function of refractive index (RI)**

We carried out simulation of the SPR as a function of RI of the surrounding media with RI ranging from 1.3154 to 1.3184, and the results are depicted in Figure S5. The enlarged drawing of the selected resonance marked with red asterisk symbols “*” is presented in Figure S5b. As we can see, the SPR is very sensitive to the change of the surrounding refractive index. Figure S5c shows a linear fit to the sensing results, and its linear regression analysis shows that the relationship between resonance intensity and surrounding refractive index (SRI) is highly linear (linearity = 99.3%) with a sensitivity of -3021.52 dB/refractive index unit (RIU).

**The in-situ monitoring system under acidic electrolyte**

As we all know, optical fiber is made of silica. It is stable in most of environments but it would be dissolved under high concentration alkaline solution. In principle, proper protection of the silica optic fiber by polymer coating might solve the issue. Alternatively using high quality plastic single mode fiber can be a solution. Both approaches can be research projects by themselves. Thus, the situation under alkaline electrolyte will not be discussed here. We mainly focus on the situation under acidic electrolyte. We selected two polypyrrole (PPy) based electrodes as energy storage materials under acidic electrolyte (1 M H_2_SO_4_). As shown in Figure S6, the SPR intensity variation can follow the CV test under different charge stored states. In addition, it can work steadily and exactly follow the charging/discharging process of the supercapacitor. Thus, these results identify the feasibility of our method under acid solution as electrolyte.

**Caption of Figures**

**Figure S1** SEM images for the gold coated fiber-optic sensing probe in different magnification.

**Figure S2** (a) Repeat cycles of CV charge/discharge test responses. (b) SPR responses for the cases of carbon fabrics with MnO_2_ and (c) without MnO_2_.

**Figure S3** CV curves of MnO_2_ based supercapacitor at different scan rates (a) 400 mV s^-1^ (e) 300 mV s^-1^ (i) 200 mV s^-1^and corresponding SPR intensity variation versus time (b), (f), (j). GCD curves of MnO_2_ based supercapacitor at different currents (c) 4 mA (g) 3 mA (k) 2 mA and corresponding SPR intensity variation versus time (d), (h), (l).

**Figure S4** Linear fits for the change of sensor SPR transmitted intensity versus the maximum stored charges under CV and GCD tests.

**Figure S5** (a) Simulation of transmission spectra of the TFBG coated with 50 nm gold as a function of SRI ranging from 1.3154 to 1.3184. (b) Enlarged drawing of the selected SPR resonance versus SRI. (c) Linear fit for the intensity of selected SPR resonance and core resonance versus SRI.

**Figure S6** (a) CV curves of the PPy based supercapacitor under different scan rates and (b) the corresponding SPR intensity variation versus time. (c) CV curves of the PPy based supercapacitor for three cycles and (d) the corresponding SPR intensity variation versus time.

**Figure S1** SEM images for the gold coated fiber-optic sensing probe in different magnification.

**Figure S2** (a) Repeat cycles of CV charge/discharge test responses. (b) SPR responses for the cases of carbon fabrics with MnO_2_ and (c) without MnO_2_.

**Figure S3** CV curves of MnO_2_ based supercapacitor at different scan rates (a) 400 mV s^-1^ (e) 300 mV s^-1^ (i) 200 mV s^-1^and corresponding SPR intensity variation versus time (b), (f), (j). GCD curves of MnO_2_ based supercapacitor at different currents (c) 4 mA (g) 3 mA (k) 2 mA and corresponding SPR intensity variation versus time (d), (h), (l).

**Figure S4** Linear fits for the change of sensor SPR transmitted intensity versus the maximum stored charges under CV and GCD tests.

**Figure S5** (a) Simulation of transmission spectra of the TFBG coated with 50 nm gold as a function of SRI ranging from 1.3154 to 1.3184. (b) Enlarged drawing of the selected SPR resonance versus SRI. (c) Linear fit for the intensity of selected SPR resonance and core resonance versus SRI.

**Figure S6** (a) CV curves of the PPy based supercapacitor under different scan rates and (b) the corresponding SPR intensity variation versus time. (c) CV curves of the PPy based supercapacitor for three cycles and (d) the corresponding SPR intensity variation versus time.
